# Achieving Excellent Dielectric and Energy Storage Performance in Core-Double-Shell-Structured Polyetherimide Nanocomposites

**DOI:** 10.3390/polym15143088

**Published:** 2023-07-19

**Authors:** You Yuan, Jingyu Lin, Xinhua Wang, Jun Qian, Peiyuan Zuo, Qixin Zhuang

**Affiliations:** Key Laboratory of Specially Functional Polymeric Materials and Related Technology (Ministry of Education), School of Material Science and Engineering, East China University of Science and Technology, Shanghai 200237, China

**Keywords:** dielectric nanocomposites, polyether imide, high temperature, dielectric properties, Fe_3_O_4_, microcapacitor structures, core-double-shell

## Abstract

The development of pulse power systems and electric power transmission systems urgently require the innovation of dielectric materials possessing high-temperature durability, high energy storage density, and efficient charge–discharge performance. This study introduces a core-double-shell-structured iron(II,III) oxide@barium titanate@silicon dioxide/polyetherimide (Fe_3_O_4_@BaTiO_3_@SiO_2_/PEI) nanocomposite, where the highly conductive Fe_3_O_4_ core provides the foundation for the formation of microcapacitor structures within the material. The inclusion of the ferroelectric ceramic BaTiO_3_ shell enhances the composite’s polarization and interfacial polarization strength while impeding free charge transfer. The outer insulating SiO_2_ shell contributes excellent interface compatibility and charge isolation effects. With a filler content of 9 wt%, the Fe_3_O_4_@BaTiO_3_@SiO_2_/PEI nanocomposite achieves a dielectric constant of 10.6, a dielectric loss of 0.017, a high energy density of 5.82 J cm^−3^, and a charge–discharge efficiency (*η*) of 72%. The innovative aspect of this research is the design of nanoparticles with a core-double-shell structure and their PEI-based nanocomposites, effectively enhancing the dielectric and energy storage performance. This study provides new insights and experimental evidence for the design and development of high-performance dielectric materials, offering significant implications for the fields of electronic devices and energy storage.

## 1. Introduction

Dielectric capacitors play a pivotal role as energy storage components in domains such as pulse power systems and electric power transmissions, owing to their exceptional attributes of ultra-fast charging and discharging rates and high power density [1,2,3]. However, the practical application of these capacitors is currently hindered by the limited energy storage density of polymer dielectrics [4]. With the growing demand for high-temperature applications, such as electric vehicles and aerospace systems, the urgent need for dielectric materials exhibiting exceptional thermal resistance has become increasingly apparent [2,5,6,7,8,9]. Therefore, the development of dielectric materials that exhibit high-temperature durability, high energy storage density, and efficient charge–discharge performance is of paramount importance. The utilization of composite materials, which amalgamate the advantages of inorganic and organic constituents, has attracted considerable attention and holds promising prospects in the electrical and electronic industries [10,11,12]. Two primary methodologies are employed in the fabrication of high-performance dielectric composites. The first approach involves the incorporation of ferroelectric ceramics possessing high dielectric constants, such as barium titanate (BaTiO_3_) [13,14], strontium titanate (SrTiO_3_) [15,16,17], titanium dioxide (TiO_2_) [18,19], and copper calcium titanate (CCTO) [20,21] into polymers. However, this method often necessitates a substantial filler content, thereby potentially compromising the mechanical properties of the composite. The second approach involves the addition of conductive fillers, such as ferroelectric oxide (Fe_3_O_4_) [22,23], graphene (GNs) [24,25,26], carbon nanotubes (CNTs) [27], MXene [28,29], etc. Among these alternatives, Fe_3_O_4_ exhibits more promising characteristics as a filler. Nevertheless, the practical application of Fe_3_O_4_ encounters certain limitations, including nanoparticle agglomeration, poor compatibility with organic matrix, and the possibility to form conductive pathways [30].

To address these challenges, coating is regarded as an effective solution that enables better filler dispersion by introducing various functional layers and thus effectively modulating the dielectric properties of composite materials. Numerous studies have confirmed the effectiveness of coating techniques [22,30,31,32,33], such as the coating of silicon dioxide (SiO_2_) on the surface of ultrafine barium titanate (BaTiO_3_) nanoparticles [34] or the coating of Fe_3_O_4_ with functionalized carbon layers and organic polyaniline (PANI) using high-temperature-resistant polymers [23]. Simultaneously, due to the limited operating temperature of commercial biaxially oriented polypropylene (BOPP) films, they fail to meet the requirements of high-temperature electrical systems [35,36,37]. Consequently, there is a compelling need to develop a dielectric material that exhibits both high energy storage density and temperature stability. Polyetherimide (PEI) holds immense promise due to its intrinsically high glass transition temperature (*T_g_* = 217 °C) and a maximum operating temperature of 200 °C, thereby satisfactorily meeting the demands of high-temperature applications [38]. By incorporating suitable fillers, the dielectric performance and energy storage density of PEI can be further enhanced while maintaining its high-temperature stability. As a result, PEI exhibits significant potential for widespread applications in high-temperature electrical systems.

Herein, we present a high-temperature polyetherimide (PEI) nanocomposite material, incorporating a core-double-shell-structured nanofiller comprising Fe_3_O_4_ nanoparticles as the core, ultrafine BaTiO_3_ ceramic as the inner shell, and insulating SiO_2_ as the outer shell. Fe_3_O_4_ nanospheres as the highly conductive core serve as the foundation for the presence of microcapacitor structures within the material. The addition of the BaTiO_3_ shell enhances the polarization and interfacial polarization strength of the composite while simultaneously acting as a barrier during the transfer of free charges. The SiO_2_ shell possesses a wide bandgap, thereby providing excellent interface compatibility and charge isolation, resulting in heightened polarization intensity, reduced dielectric loss, and diminished leakage current. The remarkable comprehensive performance of the nanocomposite can be elucidated using the interfacial polarization theory and the innovative microcapacitor structure. Under the influence of an electric field, the interfaces between the core-double-shell-structured nanofillers in the Fe_3_O_4_@BaTiO_3_@SiO_2_/PEI nanocomposite accumulate a significant amount of charge, leading to a robust interfacial polarization effect and enhanced dielectric performance. Furthermore, the regular and uniformly distributed core-double-shell-structured nanoparticles within the thin film form numerous microcapacitor structures, which effectively isolate and store charges, thereby substantially improving the dielectric and energy storage performance of the material. With a filler content of 9 wt%, the Fe_3_O_4_@BaTiO_3_@SiO_2_/PEI nanocomposite exhibits a dielectric constant of 10.6, a low dielectric loss of 0.017, and a high energy density of 5.82 J cm^−3^, with a charge–discharge efficiency (*η*) of 72%. The fabricated nanocomposite demonstrates outstanding dielectric performance, energy storage capability, and thermal stability. This innovative research provides novel insights and experimental evidence for the design and development of high-performance dielectric materials, thereby holding tremendous potential in various domains, including dielectric composites, wave-absorbing materials, energy storage materials, and supercapacitors [39,40,41,42,43,44].

## 2. Materials and Methods

Iron(III) chloride hexahydrate, ethylene glycol (AR, 99.0%), trisodium citrate dihydrate (Na_3_Cit), sodium acetate trihydrate (NaAc), cetyltrimethylammonium bromide (CTAB), and Ba(OH)_2_·8H_2_O were procured from China National Pharmaceutical Group Chemical Reagent Co., Ltd. (Shanghai, China). *N,N*-Dimethylacetamide, ethylene glycol, tetrabutyl titanate, anhydrous ethanol, acetic acid, ammonia solution (25–28%), tetraethyl orthosilicate (TEOS), and ethyl orthosilicate were obtained from Aladdin Reagent (Shanghai) Co., Ltd. (Shanghai, China). Polyetherimide (PEI, ULTEM 1000) pellets were purchased from SABIC as high-performance resin (Riyadh, Saudi Arabia). All chemicals were used without further purification.

### 2.1. Synthesis of Fe_3_O_4_ with a Hydrothermal Method

Initially, 3.25 g of FeCl_3_·6H_2_O, 1.3 g of trisodium citrate, and 6 g of sodium acetate were dissolved in 100 mL of ethylene glycol, stirring thoroughly to guarantee the complete dissolution of all compounds. Subsequently, the homogeneous solution was transferred to a 100 mL hydrothermal kettle and heated for 10 h at 200 °C. Following the reaction, the hydrothermal kettle was taken out and left to naturally cool down to room temperature. The resulting product was subsequently washed multiple times with deionized water and anhydrous ethanol to eliminate any remaining impurities and unreacted precursors. Finally, the collected sediment was placed into a vacuum oven and dried at 50 °C for 24 h, yielding Fe_3_O_4_ black powder [22,23]. 

### 2.2. Preparation of Core-Shell-Structured Fe_3_O_4_@BaTiO_3_ Nanospheres

The 0.35 g Fe_3_O_4_ nanoparticles and 0.25 g CTAB powder were weighed and uniformly dispersed in a mixture containing 100 mL of *n*-butanol and 5 mL of deionized water. The suspension was then subjected to 1 h of vigorous mechanical stirring and ultrasonication to ensure stability. Concurrently, 1.2 g of tetrabutyl titanate (TBOT) was dissolved in 50 mL of *n*-butanol, with the precursor solution formed after 1 h of continuous stirring. This solution was then gradually added to the Fe_3_O_4_ suspension under mechanical stirring and ultrasonication. Following a 24 h reaction, the products were collected, cleaned with deionized water and ethanol, and vacuum-dried to eliminate impurities and residues, resulting in Fe_3_O_4_@TiO_2_ nanoparticles. Finally, the resulting Fe_3_O_4_@TiO_2_ and 0.37 g of Ba(OH)_2_·8H_2_O were dispersed in 60 mL of deionized water and mixed thoroughly. The mixture was transferred to a 100 mL hydrothermal kettle and reacted at 200 °C for 5 h. The powder was then washed with acetic acid to eliminate BaCO_3_ impurities, and after multiple deionized water rinses and vacuum drying, Fe_3_O_4_@BaTiO_3_ nanoparticles were obtained [45].

### 2.3. Preparation of Core-Shell-Structured Fe_3_O_4_@SiO_2_ or Core-Double-Shell-Structured Fe_3_O_4_@BaTiO_3_@SiO_2_ Nanospheres 

**Figure 1:**
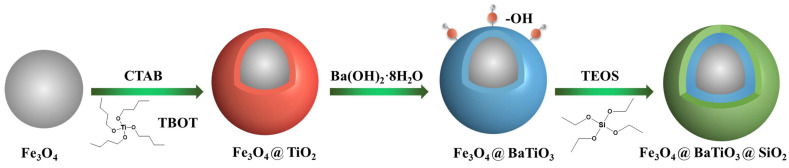
Experimental synthesis process of the core-double-shell-structured Fe_3_O_4_@BaTiO_3_@SiO_2_ nanospheres.

An aliquot of 0.15 g of Fe_3_O_4_@SiO_2_ or Fe_3_O_4_ powder was weighed and evenly dispensed into 210 mL of ethanol through ultrasonication and mechanical agitation, resulting in a stable suspension. Subsequently, 70 mL of deionized water and 4 mL of ammonia solution were introduced to the suspension and stirred at a high rate for 15 min. A precursor solution was prepared by dissolving 0.3 mL of TEOS in 10 mL of ethanol. The TEOS suspension was gradually introduced to the mixture at a pace of one drop every two seconds while maintaining continuous stirring for 12 h. Following centrifugation to collect the powder, it was rinsed multiple times with deionized water and anhydrous ethanol before being vacuum-dried to yield Fe_3_O_4_@BaTiO_3_@SiO_2_ nanoparticles. In particular, for systems of Fe_3_O_4_@SiO_2_ nanoparticles with a SiO_2_ shell thicknesses of 5 nm and 50 nm, the particle mass-to-TEOS volume ratios were 1.5 g/mL and 0.3 g/mL, respectively. The ratios of other components to particle mass remain unchanged when preparing Fe_3_O_4_@BaTiO_3_@SiO_2_ nanoparticles. Figure 1 is the schematic diagram of the preparation process of Fe_3_O_4_@BaTiO_3_@SiO_2_ nanospheres [46].

### 2.4. Preparation of PEI-Based Nanocomposite Films

The solution-casting method was employed to yield nanocomposite films with various fillers: Fe_3_O_4_/PEI, Fe_3_O_4_@BaTiO_3_/PEI, Fe_3_O_4_@SiO_2_/PEI, and Fe_3_O_4_@BaTiO_3_@SiO_2_/PEI were fabricated. PEI pellets and the four types of nanoparticles obtained were individually dispersed into DMAc through sonication for ~60 min and mechanically stirred for 4 h. Subsequently, each blend solution was subsequently cast onto a glass plate and vacuum-dried at 80 °C for 24 h in an oven, thus completely eliminating the solvent. The films were then delicately peeled from the glass plates, ultimately resulting in flexible PEI-based nanocomposite films.

### 2.5. Characterization

The nanoscale morphology of the nanoparticles was observed and analyzed using a JEOL JEM-2100 high-resolution transmission electron microscope (HR-TEM, Tokyo, Japan) and a Gemini SEM 500 energy-dispersive X-ray spectroscopy (EDS, Zeiss, Oberkochen, Germany). X-ray diffraction (XRD) analysis was conducted using a D/MAX 2550 VB/PC rotating anode X-ray diffractometer (Rigaku, Tokyo, Japan) equipped with a Cu Kα radiation source and a Ni filter (operating at 100 mA and 40 kV). Fourier-transform infrared spectroscopy (FT-IR) was performed using a Nicolet 6700 spectrometer (Thermo Fisher, Waltham, MA, USA) with the potassium bromide pellet method. Thermal gravimetric analysis (TGA) was carried out using a Netzsch TG 209 F3 Tarsus instrument from Weimar, Germany under an inert nitrogen atmosphere, with a testing range from room temperature to 800 °C. The cross-sectional images of the films were observed using a Hitachi S-4800 field emission scanning electron microscope (FESEM, Tokyo, Japan). The dielectric impedance spectra of the films were measured using a Novocontrol Concept 80 broadband dielectric impedance spectrometer in the frequency range of 10^0^ to 10^6^ Hz and a temperature range of 25–100 °C (Montabaur, Germany). According to the ASTM D149 standard [47], the dielectric breakdown strength of the films was determined using a CS2674AX high-voltage tester from Allwin Instrument Co., Nanjing, China. The polarization hysteresis loop and leakage current density were obtained using a ferroelectric polarization tester from Radiant, Inc. at a frequency of 10 Hz and at room temperature. For all electrical tests, a sputter coating technique was employed to deposit 3 mm diameter gold electrodes on both surfaces of the thin film samples. 

## 3. Results and Discussion

### 3.1. Preparation and Structure of the Nanoparticles

**Figure 2:**
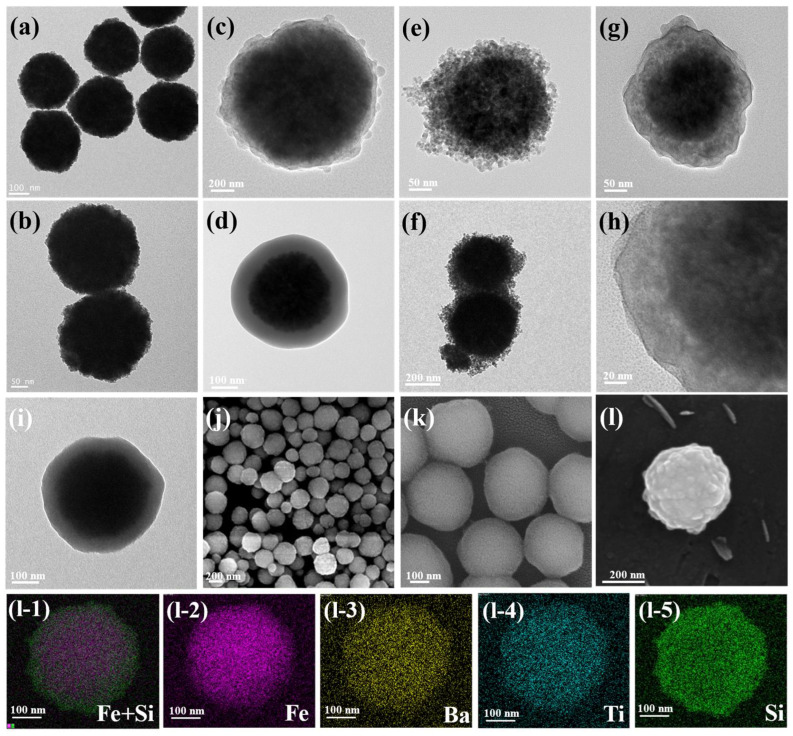
TEM images of (**a**,**b**) Fe_3_O_4_, (**c**,**d**) Fe_3_O_4_@SiO_2_, (**e**,**f**) Fe_3_O_4_@BaTiO_3_, (**g**,**h**) Fe_3_O_4_@BaTiO_3_@SiO_2_, and (**i**) Fe_3_O_4_@TiO_2_; SEM images of (**j**,**k**) Fe_3_O_4_, Fe_3_O_4_@SiO_2_ and Fe_3_O_4_@BaTiO_3_@SiO_2_; (**l**) SEM image of a single Fe_3_O_4_@BaTiO_3_@SiO_2_ particle and its (**l-1**–**l-5**) EDS images.

TEM and SEM images presented in Figure 2 and Figure S1 illustrate the structural evolution from the Fe_3_O_4_ core to the Fe_3_O_4_@SiO_2_ and Fe_3_O_4_@BaTiO_3_ core-shell structures, as well as the intermediate Fe_3_O_4_@TiO_2_, and further to the core-double-shell structure of Fe_3_O_4_@BaTiO_3_@SiO_2_. All of these nanoparticles maintain a highly spherical and uniform size. The average diameters of Fe_3_O_4_, Fe_3_O_4_@BaTiO_3_, Fe_3_O_4_@SiO_2_, and Fe_3_O_4_@BaTiO_3_@SiO_2_ are approximately 230 nm, 235/285 nm, 285 nm, and 290 nm, respectively. The silica shell thickness in Fe_3_O_4_@SiO_2_ is estimated to be around 5 nm and 50 nm (corresponding to Figure 2c,d), while the nanoscale barium titanate shell thickness in Fe_3_O_4_@BaTiO_3_ is approximately 50 nm. In Fe_3_O_4_@BaTiO_3_@SiO_2_, the respective shell thicknesses of the nanoscale barium titanate and silica are 50 nm and 5 nm. All shell thickness measurements were conducted based on Fe_3_O_4_ cores with diameters close to the average particle size (230 nm). We then used the software Digital Micrograph V3.2 to obtain a statistical average thickness.

**Figure 3:**
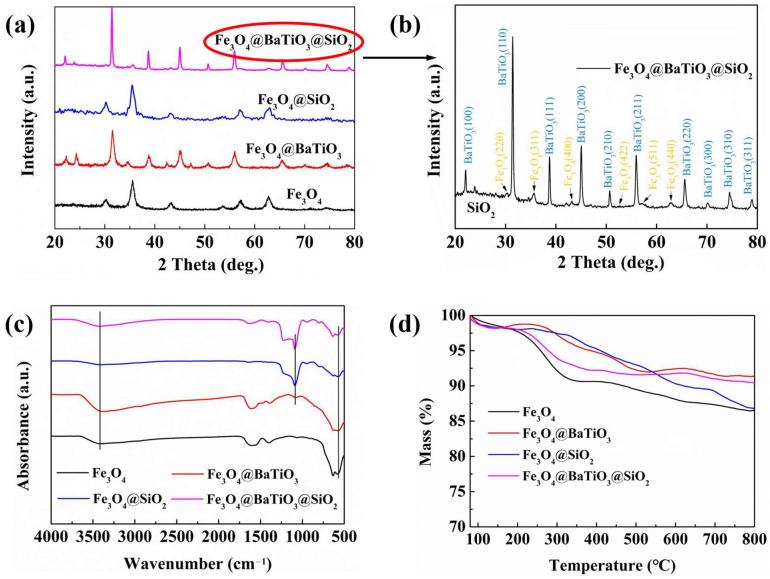
(**a**) XRD spectra; (**b**) XRD spectrum (with marked peak positions) of Fe_3_O_4_@BaTiO_3_@SiO_2_; (**c**) FT-IR spectra; (**d**) TGA curves of Fe_3_O_4_, Fe_3_O_4_@BaTiO_3_, Fe_3_O_4_@SiO_2_, and Fe_3_O_4_@BaTiO_3_@SiO_2_ nanoparticles.

**Figure 4:**
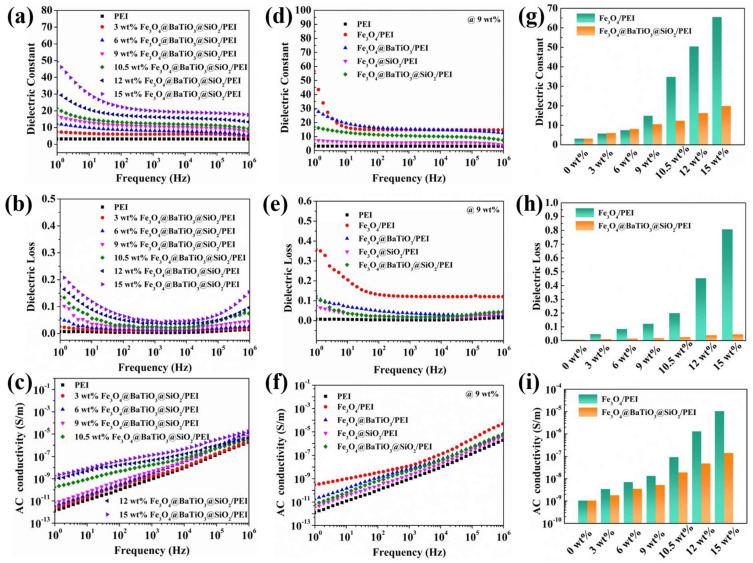
Frequency dependence of the dielectric parameters of the 9 wt% Fe_3_O_4_/PEI, Fe_3_O_4_@BaTiO_3_/PEI, Fe_3_O_4_@SiO_2_/PEI, and Fe_3_O_4_@BaTiO_3_@SiO_2_/PEI at room temperature: (**a**–**c**) are the dielectric constant, dielectric loss, and AC conductivity. Frequency dependent of (**d**) dielectric constant, (**e**) dissipation factor, and (**f**) AC conductivity of the Fe_3_O_4_@BaTiO_3_@SiO_2_/PEI nanocomposites with different filler content. Comparison of (**g**) dielectric constant, (**h**) dielectric loss, and (**i**) AC conductivity of Fe_3_O_4_/PEI and Fe_3_O_4_@BaTiO_3_@SiO_2_/PEI nanocomposites with the same filler content.

**Figure 5:**
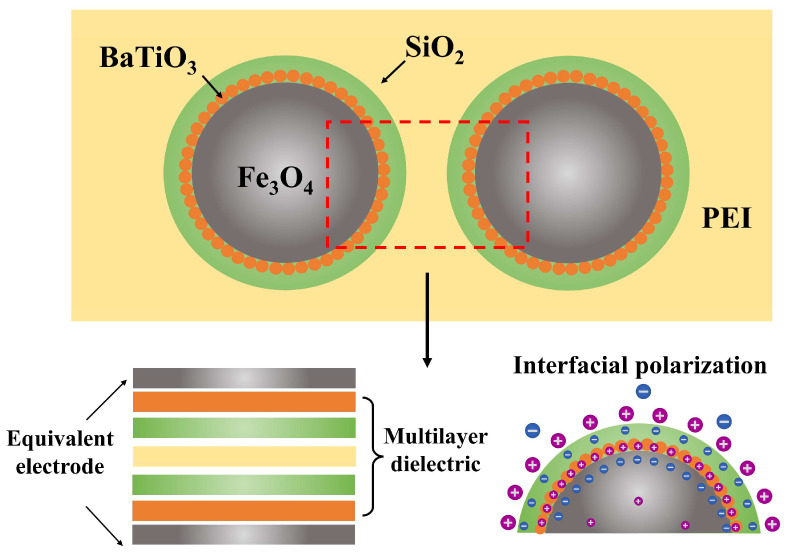
Novel microcapacitive structure and interface polarization effect within the nanocomposite thin film of Fe_3_O_4_@BaTiO_3_@SiO_2_/PEI, incorporating a core-double-shell structure for the fillers.

The EDS images shown in Figure 2(l-1–l-5) confirm the core-double-shell structure of the nanoparticles through the spatial distribution of elemental signals. The Fe elemental signal is primarily concentrated at the center of the nanoparticles, indicating the presence of the Fe_3_O_4_ core. The Ba and Ti elemental signals are predominantly concentrated around the Fe_3_O_4_ core, indicating the distribution of the BaTiO_3_ shell. Lastly, the Si elemental signal is mainly distributed on the outer surface of the BaTiO_3_ shell, indicating the presence of the SiO_2_ coating. Overlaying the Fe and Si elements provides a clearer view of the core-shell features. The elemental detection report shown in Figure S2 successfully identifies the presence of Fe, Ba, Ti, and Si in the nanoparticles. 

In the XRD spectrum of Fe_3_O_4_ (Figure 3a), there are seven prominent diffraction peaks, corresponding to the crystal planes, (220), (311), (400), (422), (511), (440), and (533), of Fe_3_O_4_, which are in accordance with the JCPDS card 01-088-0315. The X-ray diffraction (XRD) pattern of Fe_3_O_4_@BaTiO_3_ exhibits multiple distinct diffraction peaks. In addition to the diffraction peaks attributed to Fe_3_O_4_, the XRD pattern also displays seven prominent diffraction peaks assigned to the crystal planes of barium titanate (BaTiO_3_), namely (100), (110), (111), (200), (210), (211), and (220) [45]. These diffraction peaks align precisely with the crystallographic data provided by JCPDS card number 00-075-0213. Due to the amorphous nature of the encapsulated silica, lacking a long-range ordered crystal structure, the XRD spectra do not display distinct sharp peaks. Hence, the XRD patterns of Fe_3_O_4_@SiO_2_ and Fe_3_O_4_@BaTiO_3_@SiO_2_ resemble those of nanostructured Fe_3_O_4_ and Fe_3_O_4_@BaTiO_3_, respectively. The FTIR spectra of the nanoparticles shown in Figure 3c reveal a prominent absorption peak at 570 cm^−1^, corresponding to the Fe–O bond in Fe_3_O_4_. This characteristic peak is observed in all four types of nanoparticles. However, when Fe_3_O_4_ is encapsulated by the BaTiO_3_ shell and the silica shell, this feature becomes significantly weaker. The broad peak around 3410 cm^−1^ is attributed to the stretching vibration of hydroxyl groups on the nanoparticle surfaces or the absorbed water molecules. Compared to Fe_3_O_4_, Fe_3_O_4_@BaTiO_3_ exhibits a more pronounced peak at 3410 cm^−1^. This can be attributed to the smaller size and larger surface area of the synthesized barium titanate, which allows for a greater amount of hydroxyl groups and easier adsorption of water molecules. 

The FT-IR spectra of Fe_3_O_4_@SiO_2_ and Fe_3_O_4_@BaTiO_3_@SiO_2_ exhibit a strong absorption peak at 1086 cm^−1^, which is associated with the stretching vibration of Si–O–Si bonds, further confirming the presence of SiO_2_ (Figure 3c). As depicted in Figure 3d, the four types of nanoparticles exhibited no evident thermal decomposition phenomena within the tested range. The loss in mass primarily originated from the desorption of surface-adsorbed moisture or other volatile substances. Notably, all fillers displayed a residue weight exceeding 85% at 800 °C. This thermal stability serves as a fundamental prerequisite for fabricating high-temperature-resistant dielectric materials. The XPS spectrum shown in Figure S3 exhibits the elemental composition in the Fe_3_O_4_@BaTiO_3_@SiO_2_ nanohybrid filler, with all peaks corresponding to known components. 

### 3.2. Properties of the PEI-Based Nanocomposite Films

In this study, we initially embarked upon the fabrication of dielectric nanocomposites based on the principles of the percolation theory, utilizing conductive nanoparticles of Fe_3_O_4_ as the filler. As illustrated in Figure S4, the dielectric constant of Fe_3_O_4_/PEI nanocomposite films gradually increased with the increasing content of Fe_3_O_4_. At 1 kHz, the nanocomposite of Fe_3_O_4_/PEI reached its maximum dielectric constant of 65.5 when the filler content was 15 wt%, approximately 21 times higher than that of the pristine PEI film (PEI: 3.17). Furthermore, when the Fe_3_O_4_ content was below 9 wt%, the dielectric loss of the Fe_3_O_4_/PEI nanocomposite remained below 0.1. The experimental results demonstrated a remarkable change in the macroscopic physical properties of Fe_3_O_4_/PEI nanocomposite films after reaching the percolation threshold. The linear fitting of the dielectric constant and alternating current conductivity results were consistent with the predicted model (Figure S5). Although Fe_3_O_4_/PEI nanocomposite films exhibited an excellent dielectric constant, they did suffer from high losses and high electrical conductivity. These shortcomings could be mitigated by incorporating distinct functional shell layers to fabricate novel nanohybrid structures, thereby enabling precise modulation of the dielectric performance of the nanocomposite. Building upon the foundation of this study, we embarked on the synthesis of three distinct nanocomposites with core-shell and core-double-shell structures: Fe_3_O_4_@BaTiO_3_/PEI, Fe_3_O_4_@SiO_2_/PEI, and Fe_3_O_4_@BaTiO_3_@SiO_2_/PEI. Our aim was to investigate the fundamental characteristics of these materials, such as their conductivity and polarization behavior, and thus the impact of each component on the dielectric performance. By doing so, we speculated to offer valuable guidance that will further advance the design of dielectric materials.

Figure 4a–c illustrate the dielectric performance of the four nanocomposites at a filler content of 9 wt%. Fe_3_O_4_@BaTiO_3_/PEI exhibits a high dielectric constant of 14.9 at 1 kHz, which is 4.7 times higher than that of pristine PEI. The dielectric loss is reduced by 70% to 0.037 compared to the Fe_3_O_4_/PEI nanocomposite, and the AC conductivity is significantly decreased. The dielectric behavior of this nanocomposite system can be explained through an improved Maxwell–Wagner capacitor model. Initially, the introduction of ultrafine BaTiO_3_ ceramic shell layers augments the interface polarization effect between Fe_3_O_4_@BaTiO_3_ composite particles and the PEI matrix, resulting in a heightened dielectric constant of the nanocomposite. Simultaneously, due to the inherent high dielectric constant and ultra-small size of barium titanate, considerable interfacial polarization occurs between the barium titanate grains, thereby sustaining the elevated dielectric constant of the nanocomposite. Furthermore, the reduction in dielectric loss and conductivity in the Fe_3_O_4_@BaTiO_3_/PEI composite can be attributed to two factors. Firstly, the deposition of a continuous and dense layer of BaTiO_3_ ceramic on the conductive Fe_3_O_4_ surface prevents the formation of conductive pathways within the PEI matrix. Secondly, and more importantly, the numerous grain boundaries in the nano-sized BaTiO_3_ serve as traps for capturing internal free charges, making the transfer of space charges difficult. This leads to reduced energy loss, thereby suppressing dielectric loss and conductivity. The mitigation of dielectric loss is also observed in the Fe_3_O_4_@SiO_2_/PEI nanocomposite, where the dielectric constant of the 9 wt% Fe_3_O_4_@SiO_2_/PEI nanocomposite reaches 5.7 at 1 kHz, and the dielectric loss is reduced to 0.015 compared to the Fe_3_O_4_/PEI nanocomposite. This is attributed to the favorable compatibility between the SiO_2_ shell and the polymer matrix, which is crucial for achieving high-performance dielectric composites. Additionally, silica exhibits excellent insulating material that effectively inhibits charge migration and enhances the dielectric performance of the material.

Figure 4d–f illustrate the spectra of the dielectric constant, dielectric loss, and AC conductivity of Fe_3_O_4_@BaTiO_3_@SiO_2_/PEI nanocomposite films with varying component contents in the frequency range of 10^0^–10^6^ Hz. As the filler mass fraction increases from 0 wt% to 15 wt%, the dielectric constant of the Fe_3_O_4_@BaTiO_3_@SiO_2_/PEI nanocomposite rises from 3.17 to 19.90. However, this value is still lower than the dielectric constant of 65.54 observed in the Fe_3_O_4_/PEI nanocomposite under percolation effects, which is attributed to the presence of numerous microcapacitor structures within the Fe_3_O_4_/PEI nanocomposite. Interestingly, when the filler content is below 6 wt%, the Fe_3_O_4_@BaTiO_3_@SiO_2_/PEI nanocomposite exhibits a higher dielectric constant than the Fe_3_O_4_/PEI nanocomposite. This phenomenon can be attributed to the introduction of highly polarizable BaTiO_3_ nanoceramics in the Fe_3_O_4_@BaTiO_3_@SiO_2_/PEI nanocomposite at lower filling amounts, enhancing the orientational polarization of the composite. Furthermore, the double-shell structure introduces abundant interfaces, whether between the different shell layers or among the nano-sized barium titanate grains, effectively enhancing the interface polarization effect of the composite. 

In addition to the enhanced orientational polarization and interface polarization, the Fe_3_O_4_@BaTiO_3_@SiO_2_/PEI nanocomposite also contains microcapacitor structures within its internal composition. As shown in Figure 5, when we consider the conductive nanoscale Fe_3_O_4_ as the plates of a capacitor and the medium between adjacent composite nanospheres as a multilayer dielectric of the capacitor, it reveals the presence of an effective microcapacitor structure within the nanocomposite. This contributes to the excellent dielectric performance of the Fe_3_O_4_@BaTiO_3_@SiO_2_/PEI nanocomposite. The fabricated core-double-shell-structured nanocomposite exhibits outstanding dielectric properties, with a dielectric constant of 10.6 at 1 kHz and a filler content of 9 wt%, which is 3.35 times higher than that of pristine PEI. Furthermore, the dielectric loss is significantly reduced to 0.017.

As expressed using the equation $U_{e}=1/2\varepsilon_{r}\varepsilon_{0}E_{b}{}^{2}$, the breakdown strength of the dielectric is a crucial parameter that determines the working electric field and energy density. In this study, we employ the dual-parameter Weibull distribution function to analyze the dielectric breakdown behavior of both the pristine PEI and its nanocomposite films:

(1) $$P\left(E\right)=1-\exp\left[-{\left(E/E_{b}\right)}^{\beta }\right]$$

**Figure 6:**
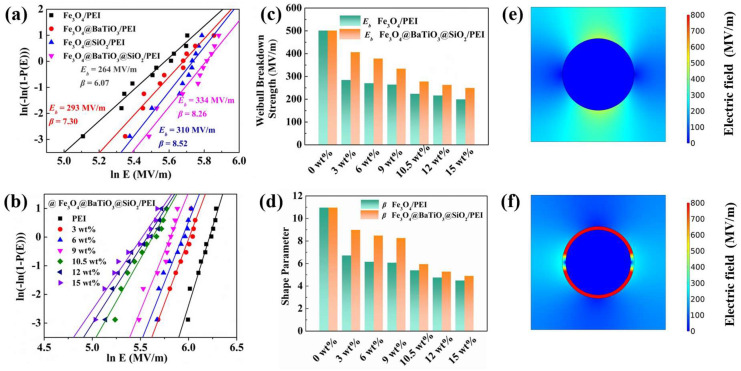
(**a**) Weibull distribution of the Fe_3_O_4_@BaTiO_3_@SiO_2_/PEI nanocomposites with different filler loadings; (**b**) Weibull distribution of the 9 wt% Fe_3_O_4_/PEI, Fe_3_O_4_@BaTiO_3_/PEI, Fe_3_O_4_@SiO_2_/PEI and Fe_3_O_4_@BaTiO_3_@SiO_2_/PEI nanocomposites; variations of (**c**) characteristic breakdown strength and (**d**) shape parameter from Weibull distribution for samples with various weight fractions of fillers; the simulation diagrams of the electric field for (**e**) Fe_3_O_4_/PEI and (**f**) Fe_3_O_4_@BaTiO_3_@SiO_2_/PEI nanocomposites.

Here, *P*(*E*) represents the cumulative probability of electrical failure, *β* denotes the shape parameter, and *E* and *E_b_*, respectively, correspond to the experimental breakdown strength and characteristic breakdown strength when the cumulative failure probability is 63.2%. A higher *β* value signifies superior film quality and exceptional structural integrity. The breakdown strength of the Fe_3_O_4_@BaTiO_3_@SiO_2_/PEI nanocomposite film is significantly improved compared to Fe_3_O_4_/PEI. Specifically, at a filler content of 3 wt%, the Fe_3_O_4_@BaTiO_3_@SiO_2_/PEI demonstrates a remarkable breakdown strength of 406 MV m^−1^, which is 1.43 times higher than that of Fe_3_O_4_/PEI nanocomposite (Figure S6) with the same filler content (284 MV m^−1^). Similarly, both Fe_3_O_4_@BaTiO_3_/PEI and Fe_3_O_4_@SiO_2_/PEI exhibit superior breakdown strength compared to Fe_3_O_4_/PEI. This can be attributed to the ability of the BaTiO_3_ shell to capture free charges and the insulating effect of the high bandgap SiO_2_ shell in isolating charges. When designing the SiO_2_ shell thickness in Fe_3_O_4_@BaTiO_3_@SiO_2_ nanoparticles, we discovered that varying the thickness of the SiO_2_ shell in Fe_3_O_4_@SiO_2_ did not significantly impact the breakdown strength of Fe_3_O_4_@SiO_2_/PEI nanocomposite films. Considering the average polarization, the thickness of the SiO_2_ shell in Fe_3_O_4_@BaTiO_3_@SiO_2_ is also designed to be 5 nm (Figure S7). Additionally, the Fe_3_O_4_@BaTiO_3_@SiO_2_/PEI nanocomposite exhibits a high *β* value (Figure 6c), indicating excellent film quality and uniformity. The 9 wt% Fe_3_O_4_@BaTiO_3_@SiO_2_/PEI nanocomposite film demonstrates a defect-free surface, with fillers uniformly distributed within the PEI matrix and displaying good interfacial interactions with the polymer (refer to Figure S8). This diminishes the non-uniformity of the electric field distribution within the composite, thereby enhancing the breakdown strength.

To better understand the advantages of the core-double-shell nanostructure in nanocomposites, we employed COMSOL Multiphysics to computationally simulate the electric field distribution in Fe_3_O_4_@BaTiO_3_@SiO_2_/PEI nanocomposite films. The simulation results indicate that in the Fe_3_O_4_/PEI nanocomposite, the surrounding region of the conductive Fe_3_O_4_ nanoparticles experiences significantly high electric field intensities. Conversely, in the Fe_3_O_4_@BaTiO_3_@SiO_2_/PEI nanocomposite, the presence of the SiO_2_ insulating shell inhibits electric field distortions near Fe_3_O_4_. The yellow areas on both sides along the electric field direction turn blue after SiO_2_ modification, indicating a greater concentration of localized electric field release.

**Figure 7:**
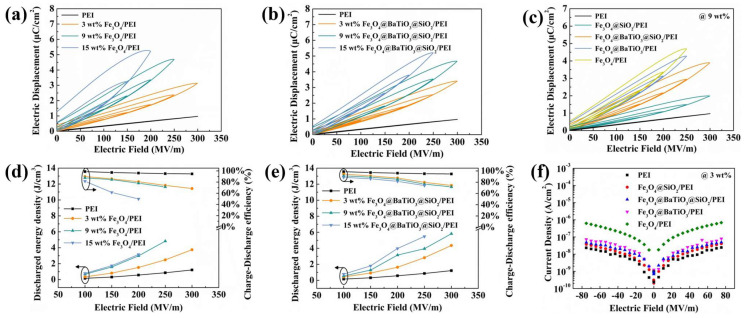
D–E loops of the (**a**) Fe_3_O_4_/PEI and (**b**) Fe_3_O_4_@BaTiO_3_@SiO_2_/PEI nanocomposites with various contents, as well as (**c**) the 9 wt% PEI-based nanocomposites; discharged energy density and charge–discharge efficiency of the (**d**) Fe_3_O_4_/PEI and (**e**) Fe_3_O_4_@BaTiO_3_@SiO_2_/PEI nanocomposites with various contents; (**f**) leakage current density of the 9 wt% PEI-based nanocomposite films under 0 to 70 MV/m electric field.

We next investigated the capacitive energy storage performance of the nanocomposites in terms of discharged energy density (*U_e_*) and charge–discharge efficiency (*η*) by referring to the unipolar D-E loops. Although the Fe_3_O_4_/PEI nanocomposite material exhibits an enhanced electrical displacement compared to pristine PEI (Figure 7a) due to the increased dielectric constant, it also results in a decrease in charge–discharge efficiency (*η*) and breakdown strength (*E*). Consequently, the energy storage performance of the Fe_3_O_4_/PEI nanocomposite film is significantly diminished. However, the fabricated core-double-shell-structured Fe_3_O_4_@BaTiO_3_@SiO_2_/PEI nanocomposite can effectively address this issue, thus allowing for the achievement of high electrical displacement (*D*, 5.21 μ cm^−2^) while maintaining a superior charge–discharge efficiency (*η*, 72%). The enhancement in electrical displacement can be attributed to the synergistic influence of orientation polarization, interface polarization, and the collaborative effect of the internal microcapacitive structure, resulting in an amplified polarization intensity. The improvement in charge–discharge efficiency (*η*) originates from the restriction of free charges with the double-shell layers, along with the low energy loss of the composite film due to the excellent compatibility between the composite nanoparticles and the matrix, ensuring minimal energy dissipation. As shown in Figure 7b,c, the Fe_3_O_4_@BaTiO_3_@SiO_2_/PEI nanocomposite film exhibits a narrower DE loop and can withstand higher test voltages. The 9 wt% Fe_3_O_4_@BaTiO_3_@SiO_2_/PEI nanocomposite achieves a high energy density of 5.82 J cm^−3^, which is an increase of 477% compared to the pristine PEI polymer under the same electric field and 120% compared to the Fe_3_O_4_/PEI nanocomposite at the maximum tested electric field. Moreover, all of the Fe_3_O_4_@BaTiO_3_@SiO_2_/PEI nanocomposites exhibit a charge–discharge efficiency (*η*) greater than 70%. Compared to similar materials such as Fe_3_O_4_@C@PANI/PBO [23], BT–Fe_3_O_4_/PVDF [48], and C@BT@R-PANI/PEI [49], the Fe_3_O_4_@BaTiO_3_@SiO_2_/PEI nanocomposite demonstrates significant advantages in terms of dielectric loss, energy density, and operating temperature (as shown in Table S1). In addition, we also investigated the leakage current density of the four nanocomposite films (Figure 7f). At 50 MVm^−1^, the introduction of Fe_3_O_4_ nanoparticles (3 wt%) reduces the leakage current from 1.24 × 10^−8^ A cm^−2^ for pristine PEI to 3.8 × 10^−7^ A cm^−2^ for Fe_3_O_4_/PEI nanocomposite material. Similarly, the leakage current density of the Fe_3_O_4_@BaTiO_3_@SiO_2_/PEI nanocomposite, with the same filler content, remains at 2.89 × 10^−8^ A cm^−2^. Both the core-shell and core-double-shell structures of the PEI composites exhibit a significant reduction in leakage current density, approximately an order of magnitude lower than that of the Fe_3_O_4_/PEI nanocomposite. This suggests that the BaTiO_3_ and SiO_2_ shell layers effectively block the conductive pathways formed by adjacent Fe_3_O_4_ nanoparticles along the electric field direction.

**Figure 8:**
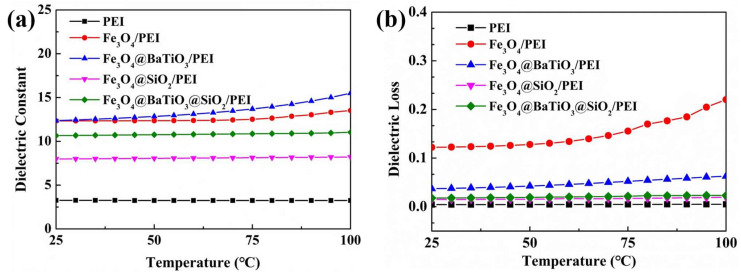
Temperature dependence of the (**a**) dielectric constant and (**b**) dielectric loss of the 9 wt% PEI-based nanocomposite films at various loadings, at 1 kHz.

In the realm of extreme application environments, the thermal stability of dielectric materials plays a significant role in assessing their long-term stability. To evaluate these properties, we conducted continuous testing of four different materials, measuring their dielectric constants and dielectric losses under a frequency of 1 kHz and temperatures ranging from 30 °C to 100 °C. The results, as depicted in Figure 8, reveal minimal variations in both the dielectric constant and dielectric loss of the Fe_3_O_4_@BaTiO_3_@SiO_2_/PEI nanocomposite material across the entire temperature range. This notable temperature insensitivity demonstrates the exceptional thermal stability of the nanocomposite material, allowing it to maintain a stable dielectric performance even in high-temperature environments (Figure S9). Such outstanding thermal stability can be attributed to both the inherent properties of the nanofillers themselves and the exceptional thermal stability of the PEI composite material. It is worth noting that the 9 wt% Fe_3_O_4_@BaTiO_3_@SiO_2_/PEI nanocomposite material exhibits a dielectric constant of 11.0 and a dielectric loss of 0.023 at 100 °C. In conclusion, these findings affirm the reliable applicability of the Fe_3_O_4_@BaTiO_3_@SiO_2_/PEI nanocomposite with its core-double-shell structure in high-temperature environments. This discovery provides crucial guidance and a solid foundation for the development of highly stable dielectric materials intended for extreme application conditions.

## 4. Conclusions

This research work has centered around the successful development, characterization, and application of the novel core-double-shell-structured nanocomposite, Fe_3_O_4_@BaTiO_3_@SiO_2_/PEI. The unique design and fabrication of this nanocomposite aim to address the limitations of conventional materials in terms of dielectric performance, energy density, and operating temperature. We began by synthesizing core-double-shell-structured nanocomposites with Fe_3_O_4_ as the core, BaTiO_3_ and SiO_2_ as the shell layers, and PEI as the matrix, using hydrothermal and solvothermal methods. The composition and morphology of the nanocomposite were validated through various analytical techniques such as XRD, TEM, and FT-IR.

By evaluating the dielectric constant, dielectric loss, and AC conductivity in the frequency range of 10^0^–10^6^ Hz with increasing filler mass fraction, we found that the Fe_3_O_4_@BaTiO_3_@SiO_2_/PEI nanocomposite demonstrates superior dielectric performance. Our results demonstrate a high dielectric constant of up to 10.6, with a dielectric loss of only 0.017 at a 9 wt% filler content, along with a significant improvement in breakdown strength compared to Fe_3_O_4_/PEI. These exceptional dielectric properties are primarily due to the polarization enhancement of the nanoceramic BaTiO_3_ and the microcapacitive structure of the multilayer dielectric capacitor. We noticed a significant improvement in the dielectric breakdown strength of the Fe_3_O_4_@BaTiO_3_@SiO_2_/PEI nanocomposite film, and the breakdown strength is 406 MV·m^−1^ at a 3 wt% filler content, which can be attributed to the BaTiO_3_ shell’s ability to capture free charges and the insulating effect of the SiO_2_ shell. Through computational simulations using COMSOL Multiphysics, we found that the SiO_2_ insulating shell in the Fe_3_O_4_@BaTiO_3_@SiO_2_/PEI nanocomposite prevents electric field distortions near Fe_3_O_4_, resulting in a higher concentration of localized electric field release.

In addition, we also investigated the energy storage performance of nanocomposite materials in terms of discharge energy density (*U_e_*) and charge–discharge efficiency (*η*). We found that the 9 wt% Fe_3_O_4_@BaTiO_3_@SiO_2_/PEI nanocomposite achieved an impressive energy density of up to 5.82 J cm^−3^, with a charge–discharge efficiency (*η*) exceeding 70%. This can be attributed to the synergistic effects of orientation polarization, interfacial polarization, and internal microcapacitor structures, as well as the confinement of free charges by the double-shell layer. Lastly, we evaluated the thermal stability of the nanocomposite. Our tests revealed minimal variations in both the dielectric constant and dielectric loss of the nanocomposite material across a temperature range from 30 °C to 100 °C, showcasing its outstanding thermal stability. Notably, even at a high temperature environment (100 °C), the 9 wt% Fe_3_O_4_@BaTiO_3_@SiO_2_/PEI nanocomposite exhibited a dielectric constant of 11.0 and a dielectric loss of 0.023, affirming its reliable applicability under extreme application conditions.

In conclusion, this study provides critical guidance and a solid foundation for the development of highly stable dielectric materials for future applications in extreme conditions.

## Data Availability

Not applicable.

## Supplementary Materials

The following supporting information can be downloaded at: https://www.mdpi.com/article/10.3390/polym15143088/s1; Figure S1: TEM image of the ultrafine barium titanate at the edges of Fe_3_O_4_@BaTiO_3_ nanoparticles; Figure S2: element detection report from the EDS analysis conducted on Fe_3_O_4_@BaTiO_3_@SiO_2_ nanoparticles; Figure S3: (a) full XPS spectrum of Fe_3_O_4_@BaTiO_3_@SiO_2_, (b–f) XPS single spectra of Fe2p, Ba3d, Ti2p, Si2p, and O1s regions, respectively; Figure S4: Frequency dependent of (a) dielectric constant, (b) dissipation factor, and (c) AC conductivity of the Fe_3_O_4_/PEI nanocomposites. (d) Variation of dielectric constant and conductivity of Fe_3_O_4_/PEI composite films with filler content; Figure S5: linear fit of (a) dielectric constant and (b) AC conductivity for Fe_3_O_4_/PEI nanocomposites (*f* ≤ *f_c_*); Figure S6: Weibull distribution of the Fe_3_O_4_/PEI nanocomposites with different filler loadings; Figure S7: Weibull distribution plots of Fe_3_O_4_@SiO_2_/PEI nanocomposite films with an SiO_2_ shell thicknesses of 5 nm and 50 nm, respectively; Figure S8: (a,b) Surface SEM images (c,d) cross-section SEM and (e,f) optical photos (focusing on the circular region in the middle) of the pristine PEI film and the 9 wt% Fe_3_O_4_@BaTiO_3_@SiO_2_/PEI nanocomposite film, respectively. (The Chinese part represents the affiliation of the authors: East China University of Science and Technology.); Figure S9: thermogravimetric curves of PEI and its nanocomposite films; Table S1: performance parameters of dielectric composites have been reported in recent years in relation to this subject. Refs. [23,45,48,49,50] are cited in the supplementary materials.

## References

[1] Zhang C., Tong X., Liu Z., Zhang Y., Zhang T., Tang C., Liu X., Chi Q. (2023). Enhancement of Energy Storage Performance of PMMA/PVDF Composites by Changing the Crystalline Phase through Heat Treatment. Polymers.

[2] Liu X.-J., Zheng M.-S., Chen G., Dang Z.-M., Zha J.-W. (2022). High-temperature polyimide dielectric materials for energy storage: Theory, design, preparation and properties. Energy Environ. Sci..

[3] Ren L., Yang L., Zhang S., Li H., Zhou Y., Ai D., Xie Z., Zhao X., Peng Z., Liao R. (2021). Largely enhanced dielectric properties of polymer composites with HfO_2_ nanoparticles for high-temperature film capacitors. Compos. Sci. Technol..

[4] Bouharras F.E., Labardi M., Tombari E., Capaccioli S., Raihane M., Ameduri B. (2023). Dielectric Characterization of Core-Shell Structured Poly(vinylidene fluoride)-grafted-BaTiO_3_ Nanocomposites. Polymers.

[5] Feng M., Feng Y., Zhang T., Li J., Chen Q., Chi Q., Lei Q. (2021). Recent Advances in Multilayer-Structure Dielectrics for Energy Storage Application. Adv. Sci..

[6] Feng Q.-K., Zhong S.-L., Pei J.-Y., Zhao Y., Zhang D.-L., Liu D.-F., Zhang Y.-X., Dang Z.-M. (2022). Recent Progress and Future Prospects on All-Organic Polymer Dielectrics for Energy Storage Capacitors. Chem. Rev..

[7] Li H., Zhou Y., Liu Y., Li L., Liu Y., Wang Q. (2021). Dielectric polymers for high-temperature capacitive energy storage. Chem. Soc. Rev..

[8] Li Y., Liu Y., Tang M., Lv J., Chen F., Li Q., Yan Y., Wu F., Jin L., Liu G. (2021). Energy storage performance of BaTiO3-based relaxor ferroelectric ceramics prepared through a two-step process. Chem. Eng. J..

[9] Lv J., Li Q., Li Y., Tang M., Jin D., Yan Y., Fan B., Jin L., Liu G. (2021). Significantly improved energy storage performance of NBT-BT based ceramics through domain control and preparation optimization. Chem. Eng. J..

[10] Bleija M., Platnieks O., Macutkevic J., Banys J., Starkova O., Grase L., Gaidukovs S. (2023). Poly(Butylene Succinate) Hybrid Multi-Walled Carbon Nanotube/Iron Oxide Nanocomposites: Electromagnetic Shielding and Thermal Properties. Polymers.

[11] Zhou W., Li T., Yuan M., Li B., Zhong S., Li Z., Liu X., Zhou J., Wang Y., Cai H. (2021). Decoupling of inter-particle polarization and intra-particle polarization in core-shell structured nanocomposites towards improved dielectric performance. Energy Storage Mater..

[12] Calvino M.M., Lisuzzo L., Cavallaro G., Lazzara G., Milioto S. (2022). Halloysite based geopolymers filled with wax microparticles as sustainable building materials with enhanced thermo-mechanical performances. J. Environ. Chem. Eng..

[13] Guo R., Luo H., Yan M., Zhou X., Zhou K., Zhang D. (2021). Significantly enhanced breakdown strength and energy density in sandwich-structured nanocomposites with low-level BaTiO_3_ nanowires. Nano Energy.

[14] Liao P., Ye H., Xu L. (2023). Improved interfacial polarization of poly(vinylidene fluoride-chlorotrifluoroethylene) composite with BaTiO_3_@polyaniline core-shell fiber. J. Appl. Polym. Sci..

[15] Chen J., Zhang X., Yang X., Li C., Wang Y., Chen W. (2021). High Breakdown Strength and Energy Storage Density in Aligned SrTiO_3_@SiO_2_ Core-Shell Platelets Incorporated Polymer Composites. Membranes.

[16] Cheng L., Liu K., Gao H., Fan Z., Takesue N., Deng H., Zhang H., Hu Y., Tan H., Yan Z. (2022). Energy storage performance of sandwich structure composites with strawberry-like Ag@SrTiO_3_ nanofillers. Chem. Eng. J..

[17] Zhang R., Sheng Q., Ye L., Long S., Zhou B., Wen F., Yang J., Wang G., Bai W. (2022). Two-dimensional SrTiO_3_ platelets induced the improvement of energy storage performance in polymer composite films at low electric fields. Ceram. Int..

[18] Yin L., Wang Q., Zhao H., Bai J. (2023). Improved Energy Density Obtained in Trilayered Poly(vinylidene fluoride)-Based Composites by Introducing Two-Dimensional BN and TiO_2_ Nanosheets. ACS Appl. Mater. Interfaces.

[19] Wang Q., Zhao H., Yin L., Ding X., Wei X., Bai J. (2022). Improved Energy Storage Performance in Sandwiched Poly(vinylidene fluoride)-Based Composites Assembling with In- Plane-Oriented BN Nanosheets and TiO_2_ Nanowires. ACS Appl. Energy Mater..

[20] Chi Q., Wang X., Zhang C., Chen Q., Chen M., Zhang T., Gao L., Zhang Y., Cui Y., Wang X. (2018). High Energy Storage Density for Poly(vinylidene fluoride) Composites by Introduced Core-Shell CaCu3Ti4O12@Al2O3 Nanofibers. ACS Sustain. Chem. Eng..

[21] Kaur S., Singh D.P. (2020). On the structural, dielectric and energy storage behaviour of PVDF-CaCu_3_Ti_4_O_12_ nanocomposite films. Mater. Chem. Phys..

[22] Fang X., Wang S., Li Y., Liu X., Li X., Lin S., Cui Z.-K., Zhuang Q. (2016). NH_2_-functionalized carbon-coated Fe_3_O_4_ core-shell nanoparticles for in situ preparation of robust polyimide composite films with high dielectric constant, low dielectric loss, and high breakdown strength. RSC Adv..

[23] Liu Q., Cheng Z., Qian J., Chen X., Zhang Y., Zhuang Q. (2019). A core@double shell-structured PBO composite with excellent dielectric properties and high heat resistance. J. Mater. Chem. A.

[24] Ni X., Feng H., Li L., Liu X., Wang T., Cui Z.-K., Gu J., Zhuang Q. (2021). A novel poly(p-phenylene benzobisoxazole) (PBO)-based three-phase silk-cocoon network structure nanocomposites with enhanced dielectric properties. J. Mater. Sci. Mater. Electron..

[25] Yuan Y., Wang X., Liu X., Qian J., Zuo P., Zhuang Q. (2022). Non-covalently modified graphene@poly(ionic liquid) nanocomposite with high-temperature resistance and enhanced dielectric properties. Compos. Pt. A-Appl. Sci. Manuf..

[26] Zhang P., Liu X., Zuo P., Mi P., Zhuang Q. (2023). Amine-Functionalized Reduced Graphene Oxide/Polyimide Nanocomposite as a Material with High Dielectric Constant and Thermal Resistance. ACS Appl. Nano Mater..

[27] Dang Z.-M., Zheng M.-S., Zha J.-W. (2016). 1D/2D Carbon Nanomaterial-Polymer Dielectric Composites with High Permittivity for Power Energy Storage Applications. Small.

[28] Tsyganov A., Vikulova M., Artyukhov D., Zheleznov D., Gorokhovsky A., Gorshkov N. (2023). Intercalation Effects on the Dielectric Properties of PVDF/Ti_3_C_2_Tx MXene Nanocomposites. Nanomaterials.

[29] Wang Z., Wu D., Kong M., Li Y., Yi Z. (2023). High energy storage density of conductive filler composites at low electric fields through sandwich design. J. Mater. Sci. Mater. Electron..

[30] Wu J.L., Zhang Y.R., Gong Y.Z., Wang K., Chen Y., Song X.P., Lin J., Shen B.Y., He S.J., Bian X.M. (2021). Analysis of the Electrical and Thermal Properties for Magnetic Fe_3_O_4_-Coated SiC-Filled Epoxy Composites. Polymers.

[31] Meng X.-S., Zhou Y., Li J., Ye H., Chen F., Zhao Y., Pan Q., Xu J. (2023). All-Organic PTFE Coated PVDF Composite Film Exhibiting Low Conduction Loss and High Breakdown Strength for Energy Storage Applications. Polymers.

[32] Lisuzzo L., Cavallaro G., Parisi F., Milioto S., Fakhrullin R., Lazzara G. (2019). Core/Shell Gel Beads with Embedded Halloysite Nanotubes for Controlled Drug Release. Coatings.

[33] Venkata Chalapathi K., Prabhakar M.N., Song J.-I. (2023). Study on the Effect of Core-Shell Abaca Vascular Carriers on the Self-Healing and Mechanical Properties of Thermoset Panels. Polymers.

[34] Bi K., Bi M., Hao Y., Luo W., Cai Z., Wang X., Huang Y. (2018). Ultrafine core-shell BaTiO3@SiO2 structures for nanocomposite capacitors with high energy density. Nano Energy.

[35] Wang Z., Meng G., Wang L., Tian L., Chen S., Wu G., Kong B., Cheng Y. (2021). Simultaneously enhanced dielectric properties and through-plane thermal conductivity of epoxy composites with alumina and boron nitride nanosheets. Sci. Rep..

[36] Wu H.H., Zhuo F., Qiao H., Kodumudi Venkataraman L., Zheng M., Wang S., Huang H., Li B., Mao X., Zhang Q. (2022). Polymer-/Ceramic-based Dielectric Composites for Energy Storage and Conversion. Energy Environ. Mater..

[37] Chen Y., Wang N., Ola O., Xia Y., Zhu Y. (2021). Porous ceramics: Light in weight but heavy in energy and environment technologies. Mater. Sci. Eng. R-Rep..

[38] Wu C., Alamri A., Deshmukh A.A., Li Z., Islam S., Sotzing G.A., Cao Y. A Modified Polyetherimide Film Exhibiting Greatly Suppressed Conduction for High-temperature Dielectric Energy Storage. Proceedings of the IEEE Conference on Electrical Insulation and Dielectric Phenomena (IEEE CEIDP) 2020.

[39] Li Z., Lin H., Ding S., Ling H., Wang T., Miao Z., Zhang M., Meng A., Li Q. (2020). Synthesis and enhanced electromagnetic wave absorption performances of Fe_3_O_4_@C decorated walnut shell-derived porous carbon. Carbon.

[40] Pei W., Shang W., Liang C., Jiang X., Huang C., Yong Q. (2020). Using lignin as the precursor to synthesize Fe_3_O_4_@lignin composite for preparing electromagnetic wave absorbing lignin-phenol-formaldehyde adhesive. Ind. Crops Prod..

[41] Chen L., Li F., Gao B., Zhou C., Wu J., Deng S., Liu H., Qi H., Chen J. (2023). Excellent energy storage and mechanical performance in hetero-structure BaTiO_3_-based relaxors. Chem. Eng. J..

[42] Wang P., Yu W., Li G., Meng C., Guo S. (2023). Printable, flexible, breathable and sweatproof bifunctional sensors based on an all-nanofiber platform for fully decoupled pressure-temperature sensing application. Chem. Eng. J..

[43] Chen L., Xu X., Wan L., Zhu G., Li Y., Lu T., Albaqami M.D., Pan L., Yamauchi Y. (2021). Carbon-incorporated Fe_3_O_4_ nanoflakes: High-performance faradaic materials for hybrid capacitive deionization and supercapacitors. Mater. Chem. Front..

[44] Qin H., Liu P., Chen C., Cong H.-P., Yu S.-H. (2021). A multi-responsive healable supercapacitor. Nat. Commun..

[45] Zhou L., Fu Q., Xue F., Tang X., Zhou D., Tian Y., Wang G., Wang C., Gou H., Xu L. (2017). Multiple Interfacial Fe_3_O_4_@BaTiO_3_/P(VDF-HFP) Core-Shell-Matrix Films with Internal Barrier Layer Capacitor (IBLC) Effects and High Energy Storage Density. ACS Appl. Mater. Interfaces.

[46] Wang Y., Sun B., Hao Z., Zhang J. (2023). Advances in Organic–Inorganic Hybrid Latex Particles via In Situ Emulsion Polymerization. Polymers.

[47] (2020). Standard Test Method for Dielectric Breakdown Voltage and Dielectric Strength of Solid Electrical Insulating Materials at Commercial Power Frequencies.

[48] Zhang C., Chi Q., Dong J., Cui Y., Wang X., Liu L., Lei Q. (2016). Enhanced dielectric properties of poly(vinylidene fluoride) composites filled with nano iron oxide-deposited barium titanate hybrid particles. Sci. Rep..

[49] Ni X., Wang X., Lin J., Liu X., Cui Z.-K., Zuo P., Zhuang Q. (2023). Ultra-low dielectric loss and high thermal stability achieved by hierarchical microcapacitor structure in nanocomposites via surface topological modulation. Mater. Today Energy.

[50] Wang H., Fu Q., Luo J., Zhao D., Luo L., Li W. (2017). Three-phase Fe_3_O_4_/MWNT/PVDF nanocomposites with high dielectric constant for embedded capacitor. Appl. Phys. Lett..

