# Supplementary material for: Achieving Excellent Dielectric and Energy Storage Performance in Core-Double-Shell-Structured Polyetherimide Nanocomposites
Source: Polymers (Basel). 2023 Jul 19;15(14):3088. doi: 10.3390/polym15143088 (PMC10386644; doi:10.3390/polym15143088)
Supplement: Supplementary file 1 [file polymers-15-03088-s001.zip › polymers-2511301-SI.pdf]

Achieving Excellent Dielectric and Energy Storage Performance in Core-Double-Shell-Structured Polyetherimide Nanocomposites

You Yuan, Jingyu Lin, Xinhua Wang, Jun Qian *, Peiyuan Zuo and Qixin Zhuang *

Key Laboratory of Specially Functional Polymeric Materials and Related Technology (Ministry of Education), School of Material Science and Engineering, East China University of Science and Technology, Shanghai 200237, China
* Correspondence: qianjun@ecust.edu.cn (J.Q.); qxzhuang@ecust.edu.cn (Q.Z.)

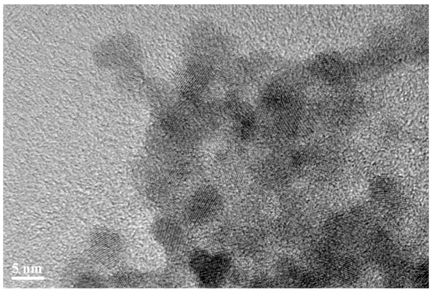

Figure S1. Experimental synthesis process of the core-double-shell structured Fe_3O_4@$BaTiO_3$@SiO_2 nanospheres.

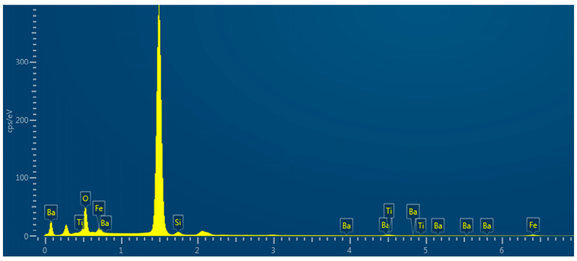

Figure S2 Element detection report from the EDS analysis conducted on Fe_3O_4@$BaTiO_3$@SiO_2 nanoparticles.

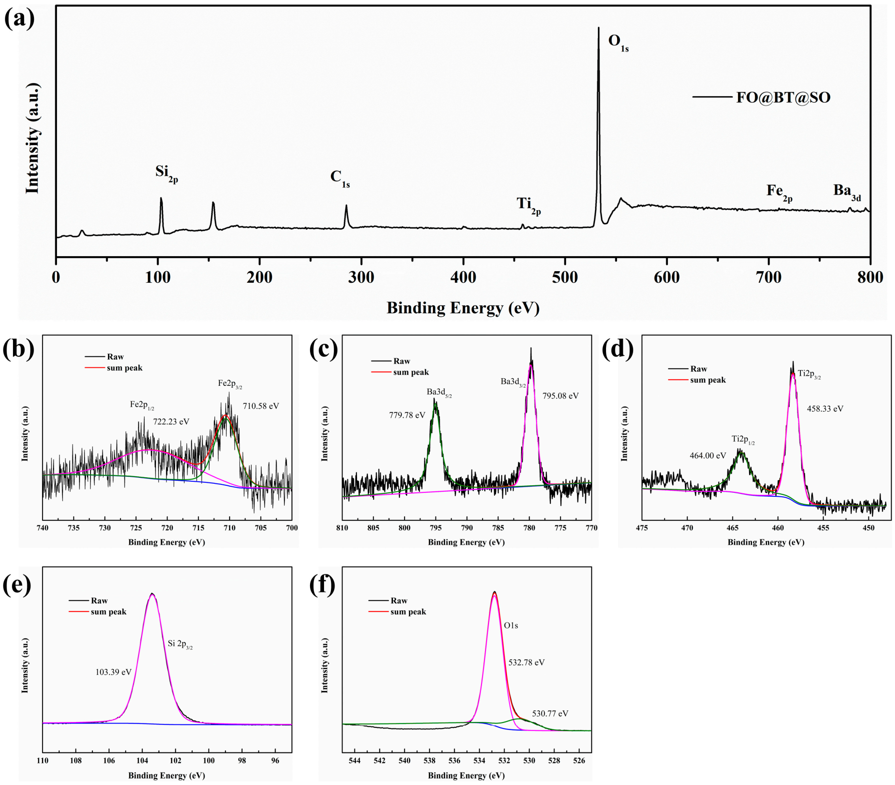

Figure S3 (a) Full XPS Spectrum of Fe_3O_4@$BaTiO_3$@SiO_2; (b), (c), (d), (e), (f) XPS Single Spectra of Fe2p, Ba3d, Ti2p, Si2p, and O1s regions, respectively.

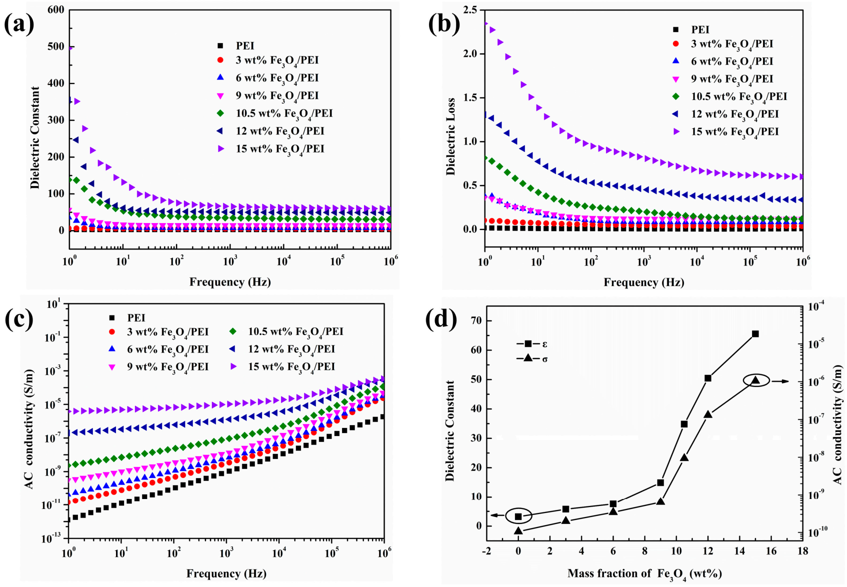

Figure S4 Frequency dependent (a) dielectric constant (b) dissipation factor and (c) AC conductivity of the Fe_3O_4/PEI nanocomposites. (d) Variation of dielectric constant and conductivity of Fe_3O_4/PEI composite films with filler content.

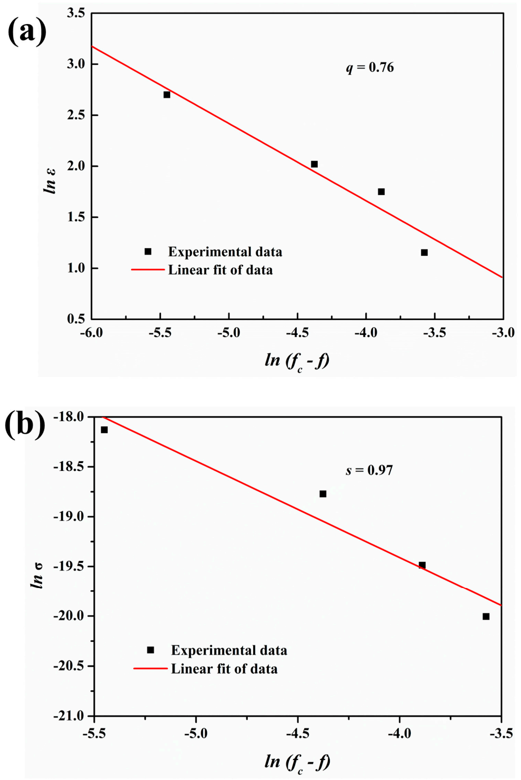

Figure S5 Linear Fit of (a) dielectric constant and (b) AC conductivity for Fe_3O_4/PEI nanocomposites ($f \leq f_c$).

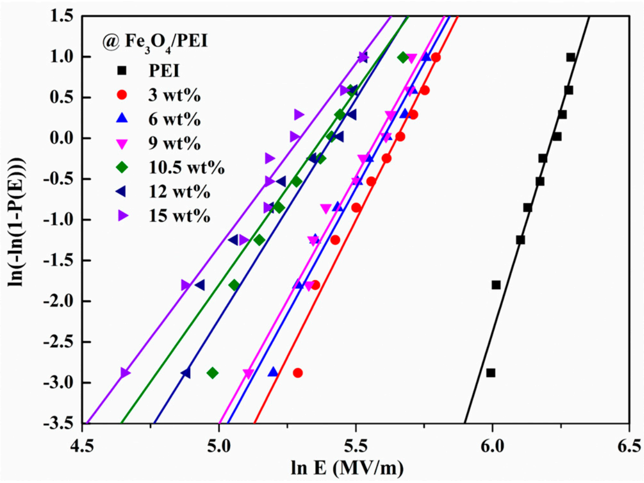

Figure S6 Weibull distribution of the Fe_3O_4/PEI nanocomposites with different filler loadings.

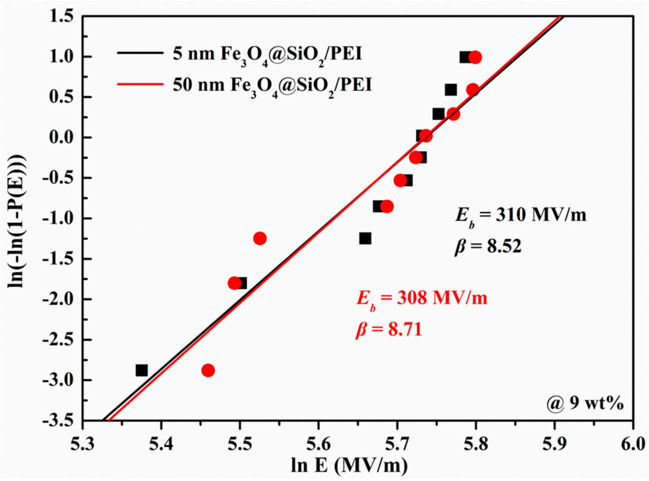

Figure S7 Weibull distribution plots of Fe_3O_4@SiO_2/PEI nanocomposite films with SiO_2 shell thicknesses of 5 nm and 50 nm, respectively.

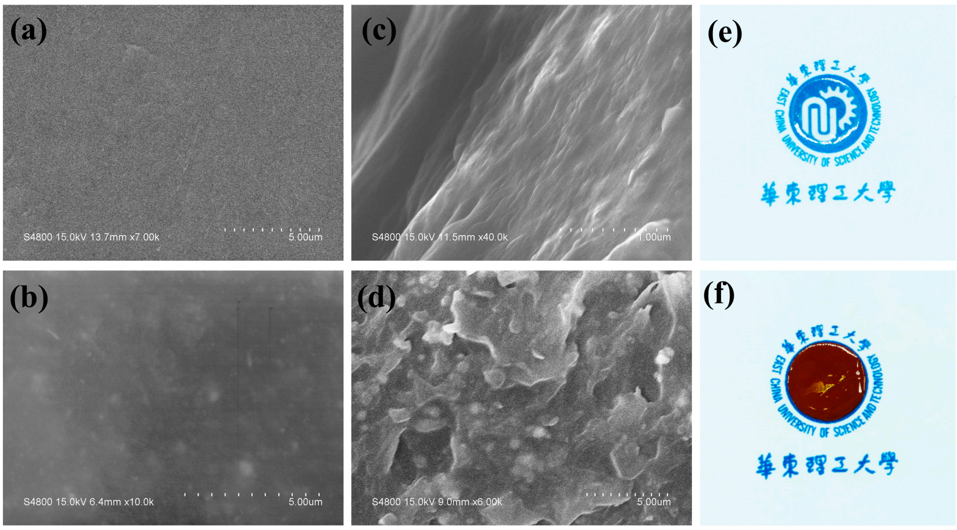

Figure S8 (a, b) Surface SEM images (c, d) cross-section SEM and (e, f) optical photos (focusing on the circular region in the middle) of the pristine PEI film and the 9 wt% Fe_3O_4@$BaTiO_3$@SiO_2/PEI

nanocomposite film, respectively. (The Chinese part represents the affiliation of the authors: East China University of Science and Technology.)

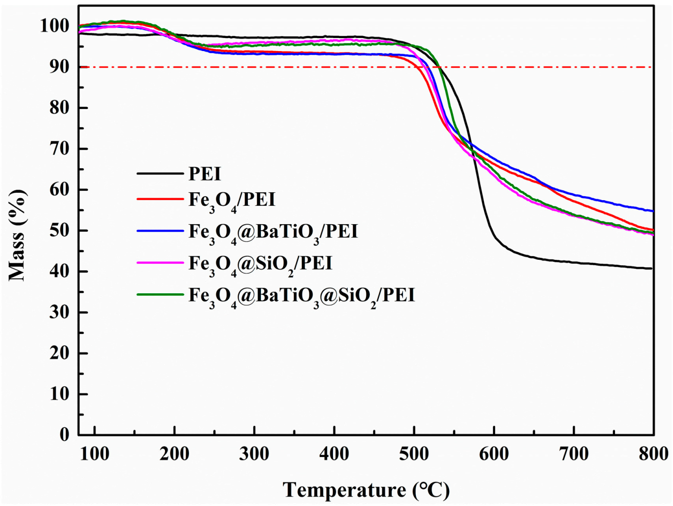

Figure S9 Thermogravimetric curves of PEI and its nanocomposite films.

Table S1. Performance parameters of dielectric composites have been reported in recent years in relation to this subject.

Filler	Matrix	Filler Content	Dielectric Loss	Discharged energy density	Test condition	Operating temperature	Ref.
Fe_3O_4@$BaTiO_3$@SiO_2	PEI	9 wt%	0.015	5.82	1 kHz	100 °C	This work
Fe_3O_4	PEI	9 wt%	0.12	4.83	1 kHz	50 °C	This work
Fe_3O_4@C@PANI	PBO	12 wt%	0.047		1 kHz	Room temperature	[1]
FO@BTO	P(VDF-HFP)	10 wt%	0.05	7.018	100 Hz	Room temperature	[2]
BT-Fe_3O_4	PVDF	40 vol%	0.24		100 Hz	Room temperature	[3]
Fe_3O_4/MWNT-S	PVDF	0.17 vol%	0.5		1 kHz	Room temperature	[4]
C@BT@R-PANI	PEI	3 vol%	0.009	2.46	1 kHz	150 °C	[5]

References

1. Liu, Q.; Cheng, Z.; Qian, J.; Chen, X.; Zhang, Y.; Zhuang, Q. A core@double shell-structured PBO composite with excellent dielectric properties and high heat resistance. Journal of Materials Chemistry A 2019, 7, 11195-11204, doi:10.1039/c8ta11796a.
2. Zhou, L.; Fu, Q.; Xue, F.; Tang, X.; Zhou, D.; Tian, Y.; Wang, G.; Wang, C.; Gou, H.; Xu, L. Multiple Interfacial Fe_3O_4@$BaTiO_3$/P(VDF-HFP) Core-Shell-Matrix Films with Internal Barrier Layer Capacitor (IBLC) Effects and High Energy Storage Density. ACS Appl. Mater. Interfaces 2017, 9, 40792-40800, doi:10.1021/acsami.7b10923.

3. Zhang, C.; Chi, Q.; Dong, J.; Cui, Y.; Wang, X.; Liu, L.; Lei, Q. Enhanced dielectric properties of poly(vinylidene fluoride) composites filled with nano iron oxide-deposited barium titanate hybrid particles. Scientific Reports 2016, 6, doi:10.1038/srep33508.
4. Wang, H.; Fu, Q.; Luo, J.; Zhao, D.; Luo, L.; Li, W. Three-phase Fe_3O_4/MWNT/PVDF nanocomposites with high dielectric constant for embedded capacitor. Applied Physics Letters 2017, 110, doi:10.1063/1.4986443.
5. Ni, X.; Wang, X.; Lin, J.; Liu, X.; Cui, Z.-K.; Zuo, P.; Zhuang, Q. Ultra-low dielectric loss and high thermal stability achieved by hierarchical microcapacitor structure in nanocomposites via surface topological modulation. Materials Today Energy 2023, 31, doi:10.1016/j.mtener.2022.101221.
